# Mechanism of Mulberry Leaves and Black Sesame in Alleviating Slow Transit Constipation Revealed by Multi-Omics Analysis

**DOI:** 10.3390/molecules29081713

**Published:** 2024-04-10

**Authors:** Chen Sun, Zheng Wang, Yang Tan, Ling Li, Feng Zhou, Shi-An Hu, Qin-Wen Yan, Lin-Hui Li, Gang Pei

**Affiliations:** 1 College of Pharmacy, Hunan University of Chinese Medicine, Changsha 410208, China; 20202095@stu.hnucm.edu.cn (C.S.); 004746@hnucm.edu.cn (Y.T.); 002181@hnucm.edu.cn (L.L.); 004871@hnucm.edu.cn (F.Z.); 201704080130@stu.hnucm.edu.cn (S.-A.H.); 20223732@stu.hnucm.edu.cn (Q.-W.Y.); 20233759@stu.hnucm.edu.cn (L.-H.L.); 2 Key Laboratory of Modern Research of TCM, Education Department of Hunan Province, Hunan University of Chinese Medicine, Changsha 410208, China; 3 Co-Construction Collaborative Innovation Center for Chinese Medicine Resources Industrialization by Shaanxi & Education Ministry, Shaanxi University of Chinese Medicine, Xianyang 712046, China; 1501001@sntcm.edu.cn; 4 National Key Laboratory Cultivation Base of Chinese Medicinal Powder & Innovative Medicinal Jointly Established by Province and Ministry, Hunan University of Chinese Medicine, Changsha 410208, China

**Keywords:** mulberry leaves and black sesame (SH), slow transit constipation (STC), transcriptomics, gut microbiota, metabolomic

## Abstract

Traditional Chinese medicine (TCM) possesses the potential of providing good curative effects with no side effects for the effective management of slow transit constipation (STC), an intestinal disease characterized by colonic dyskinesia. Mulberry leaves *(Morus alba* L.) and black sesame (*Sesamum indicum* L.), referred to as SH, are processed and conditioned as per standardized protocols. SH has applications as food and medicine. Accordingly, we investigated the therapeutic potential of SH in alleviating STC. The analysis of SH composition identified a total of 504 compounds. The intervention with SH significantly improved intestinal motility, reduced the time for the first black stool, increased antioxidant activity, and enhanced water content, thereby effectively alleviating colon damage caused by STC. Transcriptome analysis revealed the SH in the treatment of STC related to SOD1, MUC2, and AQP1. The analysis of 16S rRNA gene sequences indicated notable differences in the abundance of 10 bacteria between the SH and model. Metabolomic analysis further revealed that SH supplementation increased the levels of nine metabolites associated with STC. Integrative analysis revealed that SH modulated amino acid metabolism, balanced intestinal flora, and targeted key genes (i.e., SOD1, MUC2, AQP1) to exert its effects. SH also inhibited the AQP1 expression and promoted SOD1 and MUC2 expression.

## 1. Introduction

Slow transit constipation (STC)—an intestinal disease characterized by colonic dyskinesia—is accompanied by clinical events such as the slow transport of intestinal contents to the colon, reduced stool frequency, and stool dryness [1]. Owing to lifestyle changes with the progress in technology, issues regarding people’s quality of life, dietary conditions, and psychology are rapidly arising, accompanied by an increased incidence of constipation every year. This condition seriously affects the quality of life and physical health of people [2]. The gut barrier, gut flora, and aquaporin expression are associated with STC. In addition, patients with STC exhibit weaker antioxidant capacity and higher oxidative damage than healthy people. Intestinal damage occurs because the intestinal antioxidant stress system cannot effectively resist the increased oxidative stress [3,4,5,6,7]. Currently, Western medicine treatment for constipation promotes defecation; however, it can easily lead to complications including electrolyte disorders and intestinal nerve dysfunction owing to the long term use of medications [8]. Furthermore, it can aggravate the condition after stopping medication, resulting in drug dependency. Hence, identifying traditional Chinese medicine (TCM) preparations with good curative effects and no side effects is necessary for the effective management of STC [9].

STC pathogenesis is more complex, mainly due to the intestinal moisture loss incurred, resulting in body fluid deficiencies and affecting intestinal nourishment [10,11]. The combination of SH (mulberry leaves and black sesame) was first used in the Ming Dynasty Shoushi Baoyuan in Fusang Zhibao Dan (also known as Sangma Pill). SH has been shown to be effective in treating intestinal dryness induced by constipation.

SH has applications as food and medicine. Mulberry leaves and black sesame contain a variety of nutrients and dietary fiber, which can promote intestinal peristalsis [12,13]. Furthermore, mulberry leaf powder promotes intestinal peristalsis and water reabsorption or water-containing compound secretion, shortens defecation time, and increases the fecal moisture content in mice, exerts anti-constipation effects, and adjusts the gut microbiota [14]. Black sesame can be used to treat irritable bowel and constipation. The seeds tonify the liver and kidney and act as a moisturizing agent and laxative, which is especially suitable for patients with yin-knot-type constipation with dry intestines [15]. Research has demonstrated that SH is rich in rutin, chlorogenic acid, sesamin, and linoleic acid, all of which are capable of safeguarding the intestinal mucosal barrier and relieving symptoms associated with slow transit constipation [16,17,18,19,20,21]. Owing to the complex interactions among the components of SH, further studies should be performed to fully understand the active components and action mechanism of SH. This can be achieved by following intestinal microbiome-based and multi-omics approaches. Hence, herein, we combined intestinal microbiology, transcriptomics, and metabonomics approaches to understand the role and mechanism of SH in relieving STC, thereby providing a theoretical basis for applying TCM to treat constipation.

Ultra-performance liquid chromatography–tandem mass spectrometry was employed to determine the active components of SH. In addition, we established a mouse constipation model using loperamide to investigate the action mechanism of SH. The time of the first black stool, intestinal propulsion rate, and water content of the stool were considered the evaluation indices of constipation. The probable mechanisms were discussed from the point of view of intestinal flora composition, transcriptomics, metabonomics, intestinal mucosal barrier, aquaporins, and colon histomorphology. Additionally, molecular docking analyses were conducted to investigate the affinity for key compounds and proteins. The present results, thus, provide a theoretical basis for understanding the effect of SH on constipation.

## 2. Results

### 2.1. SH Fingerprint and Quantitative Analysis

**Figure 1:**
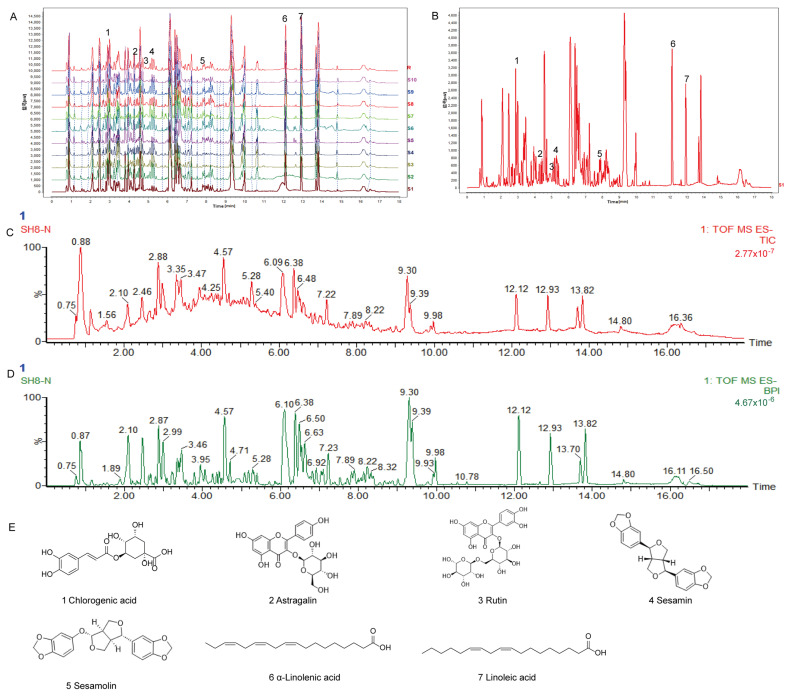
Analyzing SH with HPLC and UPLC-MS/MS. (**A**) Reproducible HPLC chromatograms from 10 batches of SH. (**B**) The total ion flow pattern and base peak pattern of SH were compared to the corresponding standards in order to identify the following 7 peaks: 1. Chlorogenic acid; 2. Astragalin; 3. Rutin; 4. Sesamin; 5. Sesamolin; 6. α-linolenic acid; 7. linoleic acid. (**C**) SH total ion flow chart. (**D**) Base peak intensity chromatogram of SH. (**E**) SH identified 7 compounds with names and structures.

Mulberry leaves and black sesame seed fingerprints were established. A total of 34 common peaks with strong absorption signals, high response values, and good stability were calibrated. The common fingerprint patterns of 10 batches of mulberry leaves and black sesame are depicted in Figure 1. The analysis of SH composition identified a total of 504 compounds (Supplementary Materials Table S1). On comparing with the corresponding reference standards, the following results were obtained: 1 was chlorogenic acid, 2 was astragalin, 3 was rutin, 4 was sesamin, 5 was sesamolin, 6 was α-linolenic acid, and 7 was linoleic acid.

SH was quantitatively analyzed by HPLC by comparing it with the corresponding standards, and chlorogenic acid (1.028 mg/g),astragalin (0.120 mg/g), rutin (0.240 mg/g), sesamin (0.837 mg/g), sesamolin (0.266 mg/g), α-linolenic acid (0.397 mg/g), and linoleic acid (2.164 mg/g) were detected (Table 1). Their chemical structures are presented in Figure 1E.

### 2.2. SH Relieves STC Symptoms in Mice

**Figure 2:**
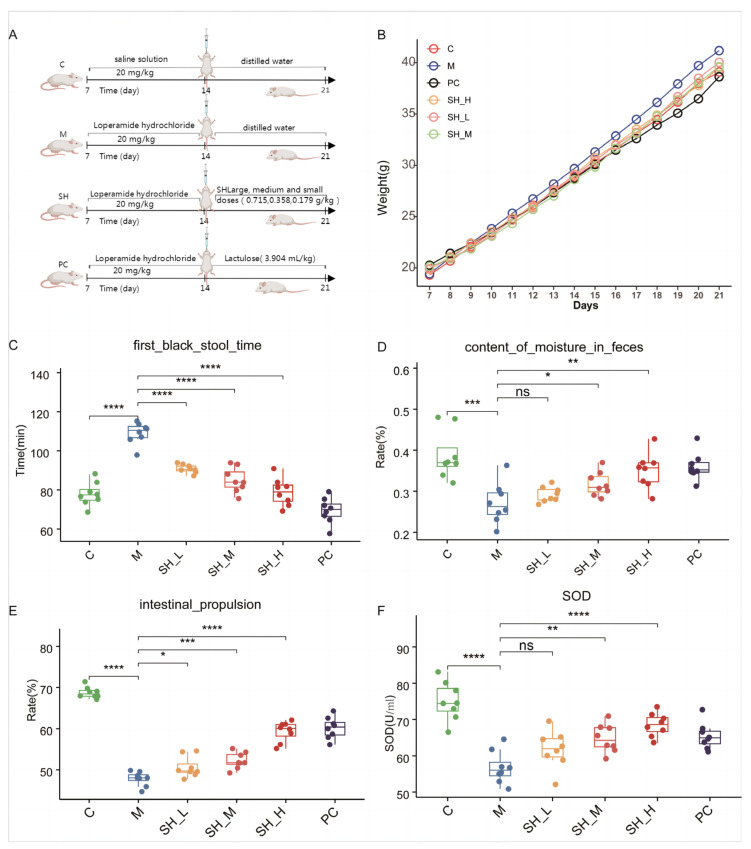
SH can alleviate the symptoms of slow transit constipation in mice. (**A**) Animal protocol; (**B**) body weight; (**C**) first black stool time; (**D**) content of moisture in feces; (**E**) intestinal propulsion rate; (**F**) SOD level. C: the control group; M: the constipation model group; SH (L, M, H): the treatment group using mulberry leaves and black sesame (low, medium, and high doses). PC group was the treatment group with lactulose. * difference between each group and the M group (* *p* < 0.05; ** *p* < 0.01, *** *p* < 0.001, **** *p* < 0.0001, ns-no significant).

SH was administered to mice with STC by gavage to study its therapeutic effect on constipation from the point of view of intestinal propulsion, shortening of defecation time, and fecal water content. Compared with the blank group, the model group showed a longer defecation period, lower water content in feces, and lower intestinal propulsion rate, whereas the STC group showed reduced feces frequency and prolonged defecation time. The SH and PC groups showed comparable efficacy. After SH intervention, compared with the model group, the STC group showed a lower defecation time, higher water content in feces, and higher intestinal propulsion rate; all these results were statistically significant (Figure 2C–E). SH significantly increases the production of antioxidant SOD (Figure 2F). SH intervention significantly promoted intestinal propulsion, shortened the time of the first black stool, enhanced antioxidant activity, and increased the water content in feces in the mice with STC. These results provide a reference for further research on developing new drugs for constipation.

### 2.3. SH Can Alleviate Colonic Injury Caused by STC

**Figure 3:**
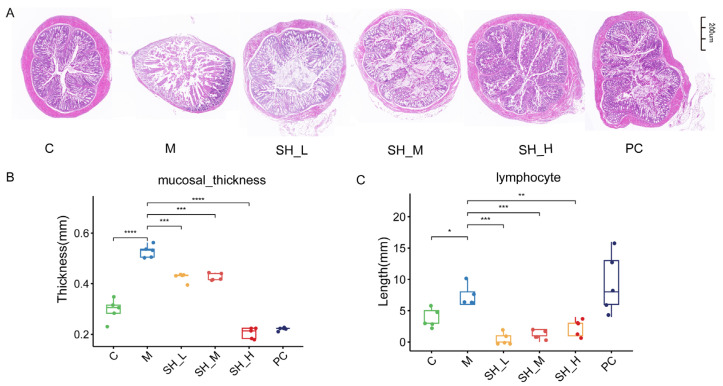
SH can alleviate colon injury in STC mice (n = 6). (**A**) Colon mucosal section HE staining (Scale bar: 200) μM, ×5). (**B**) Mucosal thickness (mm). (**C**)lymphocytes. When compared to the model group, * *p* < 0.05; ** *p* < 0.01,*** *p* < 0.001,**** *p* < 0.0001.

The colons of mice in the blank group demonstrated intact and clear structures, normal colonic mucosa, regular gland arrangement, and no abnormal or pathological changes, whereas the colons of mice in the model group showed decreased glands, irregular gland arrangement, decreased folds, flattened and shortened parts of folds, thickened mucosa, damaged muscle layers, increased lymphocyte numbers, and vacuolar degeneration in muscle cells. The SH and PC groups showed comparable efficacy. In the SH group, the aforementioned pathological changes in the colon were improved to different degrees. Thin mucosa, decreased lymphocyte numbers, and densely arranged villi were observed. Furthermore, the colon tissue injury was alleviated. Thus, SH alleviated the colonic injury caused by constipation (Figure 3). Overall, these results show that SH alleviates colon damage caused by STC.

### 2.4. Transcriptomic Analysis of SH on STC Mice

**Figure 4:**
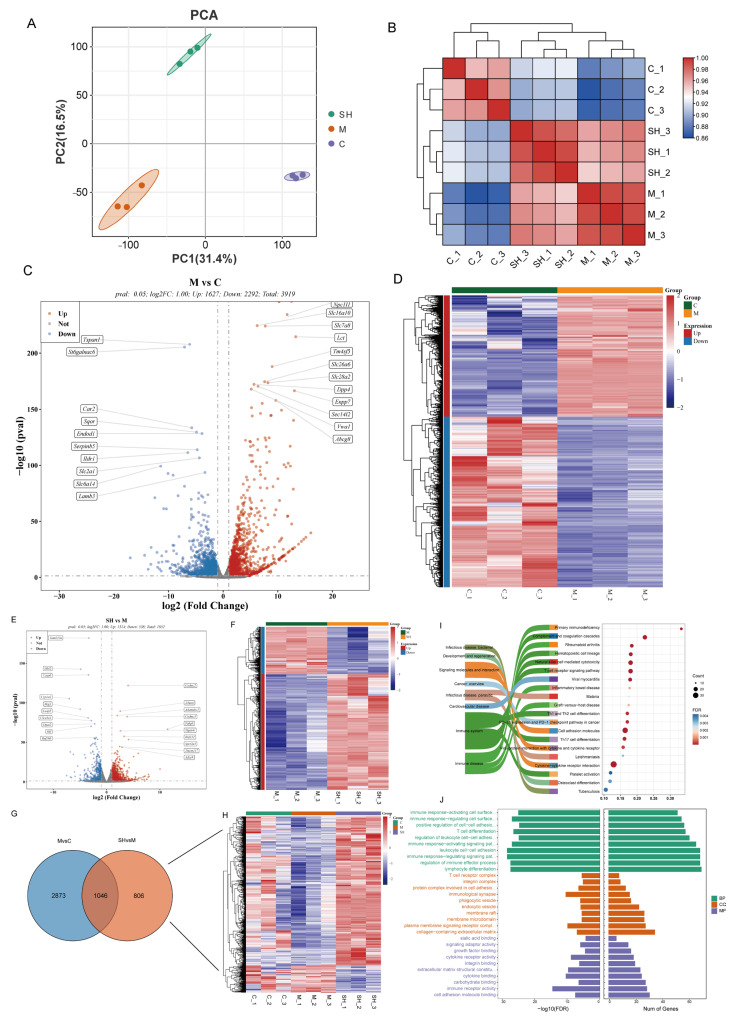
Transcriptome analysis of SH intervention in STC. (**A**) Scatter plots of groups C, M, and SH colonic transcriptome were determined by principal component analysis. Pearson’s correlational plots for (**B**); C, M, and SH correlation coefficient heat map. (**C**) In the numbers of differentially expressed genes between group C and group M, the red color indicates the upregulated genes, while the blue color indicates the downregulated genes. (**D**) Heatmaps of all differential genes between groups C and M. (**E**) Differential gene expression between groups SH and M. (**F**) SH heat map of all differential genes between group SH and group M; Venn map of (**G**,**H**) group M vs. group C and SH vs. group M; heat map of the expression of related genes in each sample. (**I**,**J**) GO enrichment analysis of SH vs. M (endemic gene).

Building upon the pharmacodynamic data, groups C, M, and SH (SH high doses) were selected for transcriptomic analysis. Principal component analysis (PCA), combined with correlation analysis between samples, revealed good repeatability within groups C, M, and SH, with significant differences between the groups (Figure 4A–D). Differential analysis between groups SH and M identified 1852 differential mRNAs, including 1314 upregulated and 538 downregulated (Figure 4E,F). To better understand SH intervention in the colon of STC mice, 806 unique differential genes related to the SH group were subjected to GO and KEGG enrichment analyses. The analysis highlighted the highest enrichment in the primary classification of Organizational Systems and Human Diseases, evidenced by the top 30 enriched GO terms and top 20 enriched pathways (*p* < 0.05) (Figure 4G–J). Furthermore, SH intervention impacted altered cytokine–cytokine receptor interaction, viral protein interaction with cytokine and cytokine receptor, Th17 cell differentiation, and Th1 and Th2 cell differentiation (Figure 4I). Transcriptome analysis revealed that SH downregulated the expression of aquaporin-related genes (AQP1-Aquaporin1) and upregulated the expression of antioxidant (SOD1-superoxide dismutase1) and intestinal mucosal barrier-related genes (MUC2-Mucin2) (Figure 4H). SH demonstrated the ability to enhance antioxidant activity, maintain the stability of the intestinal environment, and regulate the aquaporin expression.

In addition, our findings revealed that the potential therapeutic benefits of SH in treating STC may be attributed to various signaling pathways, such as cytokine–cytokine receptor interaction, viral protein interaction with cytokine and cytokine receptor, Th17 cell differentiation, and Th1 and Th2 cell differentiation. The regulation of these pathways has broad biological significance in intestinal mucosal barrier function, oxidative stress, and the expression of aquaporins.

Overall, our transcriptome analyses indicate that the synergistic effect of SH in the treatment of STC may be related to intestinal mucosal barrier function, oxidative stress, and the expression of aquaporins. This combined treatment regimen may provide essential guidance for finding novel strategies to alleviate STC.

### 2.5. 16S rRNA Sequencing Analysis of SH on STC Mice

**Figure 5:**
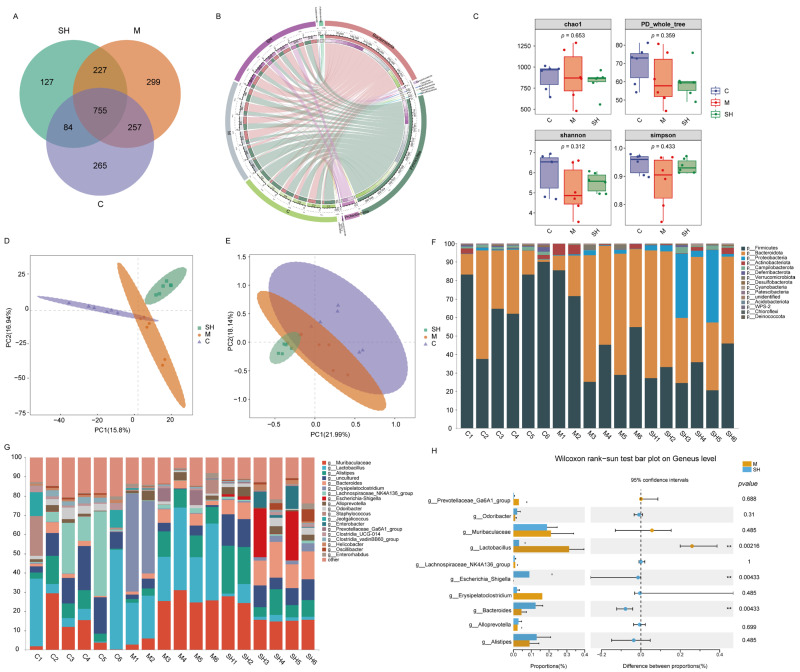
Intestinal flora analysis of SH intervention in slow transit constipation. (**A**) Venn diagram of OTUs of three groups of mouse feces samples. (**B**) Circos species relationship diagram of species composition in each sample of the microbial community in the feces samples. The outermost circle on the left depicts the sample grouping and that on the right depicts the phylum species. The innermost region represents the relative abundance percentage circle. The inner line indicates that the sample contains the species and relative abundance information. (**C**) Alpha diversity of microbiota in mouse feces during different treatments. Chao1, Simpson, Shannon, and PD_whole_tree indices were calculated for the 2014 OTUS of 9 samples. The horizontal bars within the boxes represent the averages. The top and bottom panels of the box represent the upper and lower quartiles, respectively. (**D**,**E**) PLS-DA and PCA plots. Different colors represent different groups of samples of mouse feces. The distance between points on the PCA plot represents the similarity of all samples in terms of microbiota composition and abundance. (**F**,**G**) An illustration of species composition at the phylum and genus levels. Different colors indicate different phyla and genera. (**H**) Stamp analysis of the genus level bacteria with top 10 abundance. (** *p* < 0.01).

We analyzed the intestinal flora using 16S rRNA sequencing in mice treated with mulberry leaves and black sesame, as well as the untreated control mice. The microbial composition of 18 fecal samples from groups C, M, and SH (SH high doses) was examined. Out of 1,513,702 sequences in 18 samples (average 84,094 tags), a total of 1,396,498 high-quality sequences (valid tags) were generated after quality control. At the 97% similarity level, 2014 OTUs were identified, with 755 OTUs common among the three groups (Figure 5A).

**Figure 6:**
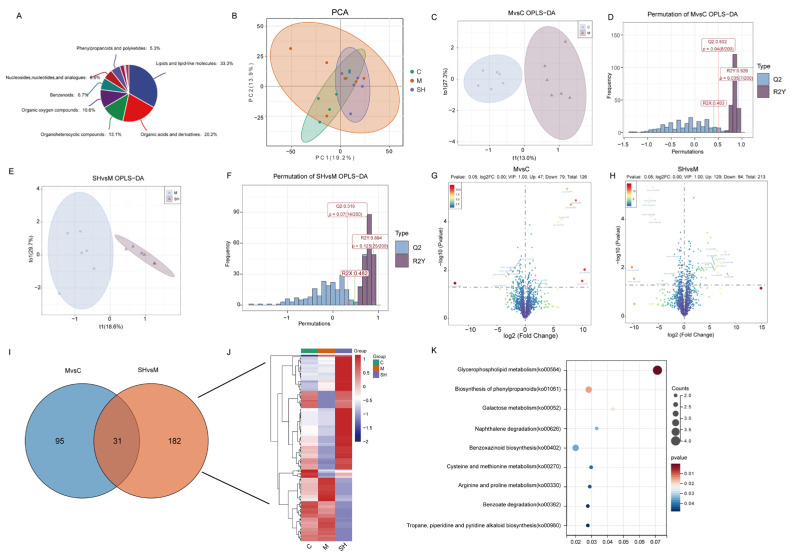
(**A**) Metabolite pie plot, (**B**) metabolite PCA analysis, (**C**–**F**) OPLS-DA plot and substitution test plot of difference combination, (**G**,**H**) volcano plot of DEMs between groups, (**I**) Venn plot between groups, (**J**) C, M, and SH thermal map, (**K**) KEGG plot of DEMs.

Initially, the Circos species relationship diagram at the phylum level (Figure 6B) revealed that groups C, M, and SH primarily contained *p_Firmicutes* and *p_Bacteroidota.* The predominant phyla observed were *p_Bacteroidota* and *p_Firmicutes*. The top five species abundance at the genus level among groups C, M, and SH were *g_Muribaculaceae*, *g_Lactobacillus*, *g_Alistipes, g_Bacteroides,* and *g_Erysipelatococcus* (Figure 5F,G). Microbiota diversity in mouse feces was evaluated using various indices, including Chao1, Simpson, Shannon, and Goods_coverage, providing insights into diversity, richness, and uniformity. The results indicated no significant differences in the four α-diversity indexes across the groups (*p* > 0.01), suggesting similar species richness and microbial uniformity (Figure 5C). However, β-diversity analysis revealed significant variations in phylogenetic distance among the three groups. PCA demonstrated distinguishability among SH, M, and C groups, indicating that different treatments could influence the overall microbial diversity of mouse feces. Additionally, partial least squares discriminant analysis identified significant differences among SH, M, and C groups (Figure 5D,E). Stamp analysis highlighted significant differences in *g_Lactobacillus*, *g_Escherichia_Shigella*, and *g_Bacteroides* between the SH and M groups at the genus level.

Further elucidating the functions of these microflora, we discovered that differentially expressed microflora may be associated with various biological processes and pathways, including intestinal motility, intestinal mucosal barrier function, and neuron development. Notably, dietary fiber-decomposing bacteria in *g_Lactobacillus*, *g_Escherichia_Shigella,* and *g_Bacteroides* may play crucial roles in intestinal mucosal barrier function.

In summary, our gut microbiota analysis revealed significant changes in the gut microbiota composition in model mice treated with SH. These findings offer valuable insights for further exploration of the mechanism underlying SH in treating STC. Future studies could concentrate on these differentially expressed flora to gain a deeper understanding of their specific mechanisms of action in the treatment of STC.

### 2.6. Metabolomics Analysis of SH on STC Mice

Transcriptomic analysis indicated that multiple metabolic pathways were involved in STC. Based on the above results, C, M, and SH groups (SH high dose) were selected for metabolic group analysis. To explore the effects of SH on metabolic changes in STC mice, non-targeted metabolomics analyses were performed. UPLC/ESI-QTRAP-MS/MS identified 1916 different metabolites in the three groups of samples, mainly concentrated in lipids and lipid-like molecules, including 331 metabolites (32.1%), organic acids and derivatives (201) (19.5%), 130 organoheterocyclic compounds 130 (12.6%), 105 organic oxygen compounds (10.2%), 67 benzenoids (6.5%), nucleosides, nucleotides, and 56 analogs (5.4%), as shown in Figure 6A. PCA analysis among C, M, and SH groups is shown in Figure 6B. Then we constructed a OPLS_DA model for MvsC and SHvsM, respectively, where R2X = 0.403R2Y = 0.926Q2Y = 0.503 for MvsC model and R2X = 0.483R2Y = 0.894Q2Y = 0.32 for SHvsM model. (OPLS_DA: Q2 indicates the prediction ability of the model, and the closer the values of R2X and R2Y are to 1, the better the fitting effect of the model.)

To explore the metabolite difference between the two groups, DEseq2 software was used to screen the metabolite difference between MvsC and SHvsM groups according to *p* value < 0.05 and vip > 2 for threshold analysis. When compared with group C, group M obtained a total of 126 differential metabolites, of which 47 differential metabolites were upregulated and 79 differential metabolites were downregulated, as shown in Figure 6G. A total of 213 differential metabolites were obtained in the SH group than in the M group, of which 129 differential metabolites were upregulated and 84 differential metabolites were downregulated, as shown in Figure 6H. In order to better study the differential metabolites related to a drug intervention in colon tissues, Venn plots were drawn for the MvsC and SHvsM groups, and 182 unique differential metabolites (DEMs) related to the drug treatment groups were obtained (Figure 6I). The expression heat map of the related metabolites in each sample is shown in Figure 6J. KEGG enrichment of specific differential metabolites was performed and SH was found to affect the following metabolic pathways: glycerophospholipid metabolism, biosynthesis of phenylpropanoids, galactose metabolism, naphthalene degradation, benzoxazinoid biosynthesis, cysteine and methionine metabolism, arginine and proline metabolism, benzoate degradation, tropane, piperidine, and pyridine alkaloid biosynthesis (Figure 6K).

The results revealed that the STC model was reliable, suggesting that the metabolism of STC model mice changed, with obvious differences among the C, M, and SH groups, while the SH group was located between M and C, indicating that the metabolic spectrum and metabolites of M group tended to be normal after SH intervention, indicating that SH intervention had a regulatory effect on metabolic disorder.

### 2.7. Combined Transcriptomics, 16S rRNA Sequencing, and Metabolomics Analysis of SH on STC Mice

**Figure 7:**
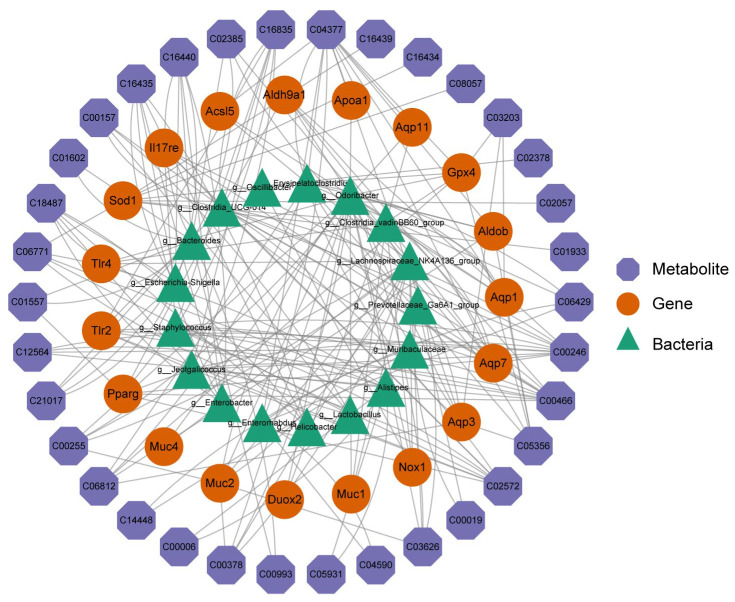
Diagram illustrating the key genes, key metabolites, and key microorganisms.

In order to further discuss the effect of SH (SH high dose) on STC mice, integrated analysis was conducted on the transcriptome, microbiome, and metabolome. Spearman’s correlational analysis was performed on the key genes, metabolites, and microorganisms using the Corrplot package. Screening was performed based on |r| > 0.6 and *p* < 0.05, and three key targets AQP1, MUC2, and SOD1 were identified (red circle in Figure 7). Twelve key metabolites were identified as 5-HPETE, 1-Hydroxy-2-naphthoic acid, 5,10-Methylenetetrahydromethanopterin, Riboflavin, and N ω, N ω- Dimethyl L-arginine, Thiamine, L-Leucine, L-Isoleucine, Cycloserine, Aminocaproic acid, L-Phenollalane, and L-Norleucine (blue hexagon in Figure 7), as well as 4 microorganisms *g__ Clostridia_ VadinBB60_ Group, g__ Odoribactor, g__ Lachnospiraceae_ NK4A136_ Group*, and *g__ Clostridia_ VadinBB60_ Group* (green triangle in Figure 7). The results are shown in Figure 7.

These results support that SH has a therapeutic effect on STC.

### 2.8. Verification of SH Core Compounds and Core Protein Targets by Molecular Docking

Furthermore, molecular docking analysis confirmed the binding affinity between the core compounds of SH (chlorogenic acid, rutin, linolenic acid, and linoleic acid) and protein targets. The identification of these core compounds in SH was achieved through LC/MS analysis. Subsequently, the experimental findings facilitated the identification of hub protein targets, namely SOD1, AQP1, and MUC2.

**Figure 8:**
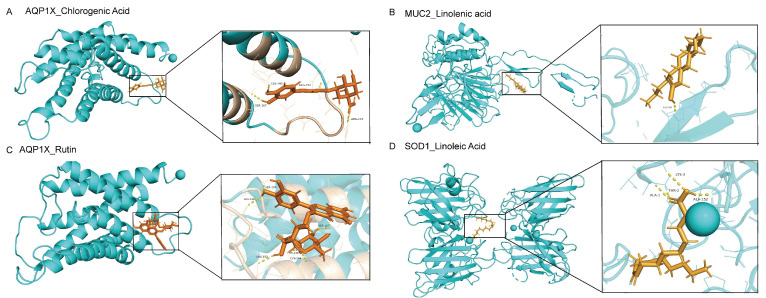
(**A**) Molecular docking diagram of AQP1 vs. chlorogenic acid, (**B**) MUC2 with Linolenic acid, (**C**) AQP1 vs. Rutin, and (**D**) SOD1 vs. linoleic acid.

As presented in Table 2, the binding affinity of the four core compounds to the protein crystal structure corresponding to the core target gene exceeded −5 kcal/mol, indicating a substantial affinity of the compounds toward the protein crystal structure. Particularly, AQP1 exhibits an affinity of −6.952 kcal/mol toward chlorogenic acid and that of −8.183 kcal/mol toward rutin. MUC2 demonstrates a notable binding affinity of −7.277 kcal/mol with linolenic acid. Similarly, SOD1 exhibits a considerable affinity of −7.047 kcal/mol toward linoleic acid. As depicted in Figure 8 and Table 2, the tight binding of small molecule compounds to protein residues was facilitated via diverse interactions.

### 2.9. SH Inhibited AQP1 Expression and Promoted SOD1 and MUC2 Expression

**Figure 9:**
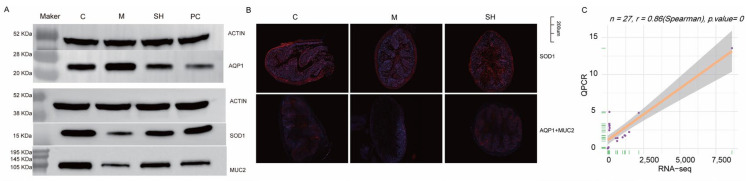
SH inhibited the AQP1 expression and promoted the SOD1 and MUC2 expression (*n* = 3). (**A**) Western blotting was performed to detect the protein expression of AQP1, MUC2, and SOD1 in the colon tissues. (**B**) Representative images of AQP1, MUC2, and SOD1 immunofluorescence in mouse colon tissues (Scale bar: 200 μM, × 5). (**C**) QPCR validation results.

To assess the impact of SH on oxidative stress, aquaporin, and MUC2, we scrutinized the expressions of SOD1, AQP1, and MUC2. The results revealed that AQP1 was widely distributed in the mice colon tissues of the model group, exhibiting increased expression, whereas the expressions of SOD1 and MUC2 were decreased. Following SH intervention, the expressions of SOD1 and MUC2 recovered, whereas AQP1 expression decreased after SH treatment. These findings indicate that SH could effectively inhibit oxidative stress, regulate aquaporins, and preserve the integrity of the intestinal mucosal barrier. Correlation = 0.8, *p* < 0.05, the results of qpcr, and transcription are consistent. Figure 9 shows the experimental results.

Altogether, our results suggested that SH has the potential to increase the expression of SOD1 and MUC2 while reducing that of AQP1. These findings lay a crucial foundation for future research on the biological significance of SH and its potential utility in treating STC.

## 3. Discussion

The medicinal combination of “mulberry leaves and black sesame” has historical roots dating back to the Ming Dynasty’s “Shoushi Baoyuan” in Fusang Zhibao Dan (also known as Sangma Pill), renowned for its efficacy in treating constipation caused by dryness in the intestines. Our study substantiates this historical knowledge by demonstrating significant improvements in the symptoms of STC and stool hardness with the administration of SH. Notably, the time to the first black stool was reduced, and the intestinal propulsion rate and fecal water content increased (Figure 1C–E). These results underscore the potential of SH to enhance intestinal motility, expedite stool passage, and increase water absorption in mice with STC. Lactulose is a common mild laxative often used to treat slow transit constipation. In this experiment, lactulose served as the positive control drug and was compared with SH, showing comparable efficacy in alleviating STC. Nevertheless, further research is warranted to investigate the broader applicability of this combination to other forms of constipation and to elucidate its specific action mechanisms.

The pathogenesis of STC is intricate, with intestinal motility deficiency identified as a key pathological mechanism. Intestinal motility includes various physiological aspects, including contractile movement of the intestinal muscles, sensitivity of the intestinal wall, biomechanical function of the intestinal tract, and colon injury [22]. Our findings suggest a potential role for SH in mitigating constipation-induced colonic injury. These substances may contribute to colon health by reducing the number of lymphocytes, promoting the return of villous epithelium to normal length, and improving the thickness of colonic mucosa. While these results provide a foundation for further exploration and the application of this combination in mitigating constipation-related colonic pathological damage, additional research is necessary to comprehend the underlying action mechanism and explore the potential clinical applications.

The interaction between cytokines and their receptors, along with the interplay between viral proteins and cytokines and cytokine receptors, correlates with antioxidant activity. Research highlights the significance of certain cytokines and their receptors in regulating oxidative stress and responding to antioxidants. Distinct signaling pathways of cytokines govern the modulation of antioxidant enzyme expression and activity, as well as the expression of genes associated with oxidative stress. Moreover, some cytokines can directly influence the balance of free radical production and clearance [23]. Viral infections often trigger inflammatory responses, and viral proteins can interact with cytokines and their receptors, disrupting normal cell signaling. This disruption may weaken intracellular antioxidant defenses, intensify oxidative stress, and induce cellular and tissue damage [24]. Further studies are needed to elucidate the specific interaction and regulatory mechanisms in these pathways.

A close relationship exists between Th17 cell differentiation and the intestinal mucosal barrier. Th17 cells, specific immune cells, play a crucial role in regulating immune balance and maintaining the integrity of the intestinal mucosal barrier during immune responses. The intestinal mucosal barrier, formed through the collaboration of intestinal epithelial cells and mucosal immune cells, protects the intestinal tract from harmful substances and microorganisms [25]. Th17 cells, by secreting cytokines such as interleukin (IL)-17A and IL-22, promote the production of mucus, including MUC2, by intestinal epithelial cells. This event enhances adhesion and strengthens the integrity of the intestinal epithelial layer [26]. Th17 cell secretions also assist mucosal immune cells, such as macrophages and dendritic cells, in activating and guiding immune responses to maintain immune balance. The precise regulation of the relationship between Th17 cells and the intestinal mucosal barrier is an ongoing area of study influenced by numerous factors.

The results suggest a potential correlation between the activity and secretion of Th1 and Th2 cells and the expression and regulation of aquaporins. Past studies indicate that Th1 cell-secreted IFN-γ can impact the expression of aquaporins, particularly AQP1 [27]. Conversely, Th2 cell-secreted cytokines IL-4 and IL-13 may increase the expression and function of aquaporins, with a notable impact on AQP1 [28]. This relationship may be associated with the role of Th2 cells in anaphylaxis and immune regulation. However, it is essential to note that the association between Th1 and Th2 cell differentiation and aquaporins requires further in-depth analysis and verification, particularly in the context of STC.

*Lactobacillus*, *Escherichia_Shigella*, and *Bacteroides*, which are the common members of intestinal flora, exhibit relationships with constipation [29]. *Lactobacillus*, a probiotic class, plays a pivotal regulatory role in gut health. It contributes to balancing intestinal flora, enhancing intestinal motility, and promoting defecation, thereby aiding in preventing constipation. *Lactobacillus* is closely associated with the intestinal mucosal barrier, participating in its maintenance and promotion by enhancing adhesion, producing antibacterial substances, regulating immune responses, and aiding in repair. The relationship between *Lactobacillus* and aquaporins involves maintaining the acid–base balance of the intestinal microenvironment and regulating water transport across intestinal epithelial cells [30]. Some studies have indicated that certain *Lactobacillus* strains exhibit high antioxidant enzyme activity, such as superoxide dismutase (SOD), thereby contributing to the clearance of free radicals and acting as antioxidants [31].

*Escherichia_Shigella*, a type of intestinal bacteria, has been associated with stimulating intestinal peristalsis and water absorption, potentially preventing constipation through the production of short-chain fatty acids (SCFAs), such as propionic and acetic acids. However, it is noteworthy that *Escherichia_Shigella* may be linked to intestinal diseases and digestive issues in some cases, potentially impacting bowel functions [32,33].

*Bacteroides*, another group of intestinal probiotics, play a crucial role in maintaining the ecological balance of the intestine. Past studies suggest that *bacteroides* abundance is associated with normal intestinal motility and defecation. Being involved in the production and maintenance of the intestinal mucus layer, *bacteroides* contribute to protecting the intestinal mucosa [34]. By decomposing substrates such as dietary fiber, *bacteroides* produce metabolites, including SCFAs, thereby supporting the growth and health of the mucus layer, which, in turn, helps alleviate STC [35].

Research indicates that the balance of intestinal flora is closely linked to the function of aquaporins. An imbalance in the intestinal flora may result in the abnormal expression or functional impairment of aquaporins, potentially affecting the normal passage of water molecules. While Odoribacter, Clostridia vadinBB60 group, and AQP1 may not be directly related to biological function, the composition and function of the gut microbes are intricately connected to the health of the host. Disturbances in intestinal microbiota have been associated with the occurrence and development of STC, and aquaporin AQP1 may also play a role in this context [36].

There exists an intricate interaction relationship between Lachnospiraceae_NK4A136_Group, Odoribacter, and MUC2. Past studies have revealed that Lachnospiraceae_NK4A136_Group can positively influence the production and secretion of MUC2, thereby enhancing the protective function of the mucosal barrier [37]. Moreover, the degradation products of MUC2 can serve as nutrients for Lachnospiraceae_NK4A136_Group, promoting its growth and reproduction. Odoribacter, on the other hand, is involved in the degradation of dietary fiber and oligosaccharides, producing SCFAs. These fatty acids contribute to the expression and secretion of MUC2, leading to increased production of the mucus layer [38]. Additionally, Odoribacter generates metabolites, including butyric acid and acetic acid, which can directly or indirectly regulate the expression of MUC2. The reciprocal relationship between Odoribacter and the integrity of the mucus layer is pivotal for maintaining intestinal health and balance.

Research indicates that gut microbiota plays a crucial role in influencing the antioxidant capacity of the body. The dysregulation of certain intestinal flora has been associated with a decrease in the SOD1 activity. Changes in the intestinal flora may lead to the downregulation of the SOD1 gene, thereby impairing the ability of the cell to scavenge superoxide radicals. Members of the Clostridia_vadinBB60_group may produce metabolites, such as SCFAs, which can affect the activity and expression of SOD1. Certain SCFAs have been shown to promote the expression and activity of SOD1, thereby enhancing the antioxidant capacity of cells [39,40].

The integrated analysis of transcriptome, microbiome, and metabolome revealed that SH primarily impacted amino acid metabolism, suggesting that deficiencies or abnormal metabolism of amino acids could potentially be associated with constipation [41]. In addition, the interaction between amino acid metabolism and intestinal flora also plays a certain role in constipation. *g__Clostridia_vadinBB60_group, g__Odoribacter*, and *g__Lachnospirace_NK4A136_group* are closely related to amino acid metabolism [42]. Based on the correlation results mentioned above, three targets were identified: SOD1, AQP1, and MUC2. STC has a close association with aquaporins, and it has been found that SH can downregulate AQP1, which aligns with the findings reported in the literature [43,44]. Abnormal expression of aquaporins can induce an oxidative stress response, and SH can upregulate SOD1, which is consistent with the literature reports [45,46]. Oxidative stress response has the potential to induce damage to the intestinal barrier, with MUC2 serving as a crucial marker for its integrity. SH can enhance the secretion of MUC2, thus effectively mitigating STC, which aligns with the existing literature findings [47,48]. This study reveals the upregulating effects of SH on SOD1 and MUC2, as well as its downregulating effects on AQP1, highlighting the potential regulatory roles of these targets.

## 4. Materials and Methods

### 4.1. Reagents and Materials

The raw materials for SH, that is, 125 g of mulberry leaves and 30 g of black sesame, were purchased from Shaanxi Xingshengde Pharmaceutical Co., Ltd. (Xi’an, China). Loperamide was purchased from Shanghai Yuanye Biotechnology Co., Ltd., Shangai, China (batch number: A3ois224196). Saline for injection was purchased from Sichuan Kelun Pharmaceutical Co., Ltd., Sichuan, China (batch number: H20083400). The lactulose oral solution was purchased from Beijing Hanmei Pharmaceutical Co., Ltd., Beijing, China (Batch No. 22120023). India ink (biological dye) was purchased from Shanghai Source Leaf Biotechnology Co., Ltd., Shangai, China (batch number: A3ois224570). MUC2 (Servicebio, Wuhan, China, No. GB12345), SOD1 (Servicebio, No. GB112451), AQP1 (Servicebio, No. PB9473), RIPA lysate (Servicebio, No. G2002-100ML), the BCA protein quantitative detection kit (Servicebio, No. G2026). Additionally, the following chemical was purchased from the respective manufacturer: chlorogenic acid (purity >98%, Shanghai Yuanye Biotechnology Co., Ltd., batch number A22GB158496), rutin (purity >98%, Shanghai Yuanye Biotechnology Co., Ltd., batch number J01IB203749), Astragalin (purity >98%, Shanghai Yuanye Biotechnology Co., Ltd., batch number F18HB175834), Sesamin (purity >98%, Shanghai Yuanye Biotechnology Co., Ltd., batch number C16D10H105983), Sesamolin (purity of 98%, Shanghai Yuanye Biotechnology Co., Ltd., batch number A15GB157723), α-linolenic acid (purity >98%, Shanghai Yuanye Biotechnology Co., Ltd., batch number J13IB220095), and linoleic acid (purity >98%, Shanghai Yuanye Biotechnology Co., Ltd., batch number M21HB178273).

### 4.2. Animal Experiments

An ethics committee at Shaanxi University of Chinese Medicine approved all animal experiments (SUCMDL20211010023). These experiments were conducted in accordance with the 1985 revised guidelines for the Care and Use of Experimental Animals (NIH publications 85-23). We used specific pathogen-free Kunming mice (KM; male, 18 ± 2 g) purchased from Chengdu Dasuo Experimental Animal Co., Ltd., Chengdu, China. During the experiments, the temperature was maintained at 24 ± 2 °C, the humidity was maintained at 50 ± 5 °C, the mice were confined to an area of 18 m^2^, and the light/dark cycle lasted for 12 h. A total of 72 KM mice were fed for 7 days before the experiment and randomized into six groups, namely the control group, model group (loperamide), mulberry leaves–black sesame seed group (SH) low-dose, SH medium-dose, SH high-dose, and lactulose group (*n* = 12 in each group). Except for the control group, the remaining groups were administered loperamide hydrochloride (20 mg/kg) by gavage (once a day) for seven consecutive days to establish the STC model. In contrast, the control group was given distilled water by gavage (20 mg/kg once a day). The doses administered to mice in the treatment groups were as follows: SH-L (0.179 g/kg), SH-M (0.358 g/kg), SH-H (0.715 g/kg), lactulose group (3.904 mL/kg), and the model group was given distilled water of the same dose. These doses were administered once a day for 7 consecutive days. The mice were weighed daily, and their serum and colon tissues were collected and stored in liquid nitrogen at the end of the experiment.

### 4.3. Studies on the Fingerprint of SH

SH extract (0.1 g) was accurately weighed and placed in a conical flask with a stopper. After adding methanol (20 mL), the solution was weighed and sonicated (power: 300 W; frequency: 40 kHz) for 30 min. Further, the solution was weighed again after cooling down. More methanol was added to compensate for the loss of weight, and the solution was shaken thoroughly, followed by filtration to obtain the filtrate. Next, the precision, stability, and repeatability experiments, fingerprint establishment, similarity evaluation, cluster analysis, and PCA of the mulberry leaves–black sesame seed solution (10 batches) were performed. The following instruments were used for the experiments: the Acquity UPLC System (Waters, Milford, MA, USA), the Synapt G2-Si Q-TOF High Definition MS System mass spectrometer (Waters), and the Masslynx V4.1 workstation (Waters). Chromatographic conditions were as follows: column ACQUITY UPLCTM Beh C18, 2.1 × 100 mm, 1.7 μm; mobile phase, 0.1% formic acid aqueous solution: (a) acetonitrile; (b) gradient elution; flow rate, 0.3 mL/min; column temperature, 40 °C; sample size, 1 mL.

### 4.4. Quantitative Analysis and Detection of SH Using High-Performance Liquid Chromatography (HPLC)

An accurately weighed amount containing 130 mg of mulberry leaves and sesame extract was placed in a 5 mL volumetric flask with a stopper. Then, 4 mL of methanol was added to it, and the flask was sealed. The solution was subjected to ultrasound treatment at 25 °C (power 600 W, frequency 60 kHz) for 30 min and then placed at 25 °C. Next, methanol (4 mL) was added to the mark, and the solution was shaken well and filtered through a 0.22 μM membrane to obtain the desired filtrate. Accurately weighed (10 mg) chlorogenic acid, rutin, astragalin, sesamin, and sesamolin were mixed with 10 mg of α-linolenic acid and 10 mg of linoleic acid in a 10-mL volumetric flask. The mixture was dissolved in 1 mL of dimethyl sulfoxide, and then the final volume (10 mL) up to the mark was achieved by adding the 50% methanol aqueous solution. The reserve solution had a reference substance concentration of 1 mg/mL. Chromatographic conditions (Vanquish Flex Thermo Fisher Scientific, Waltham, MA, USA) were as follows: Acclaim RSLC C18 column, 100 mm × 2.1 mm, 2.2 μm; mobile phase, A phase: 0.1% formic acid aqueous solution and B phase: methanol; gradient elution, 0–1 min, 10% B and 90% A; 1–2 min, 10–20% B and 90–80% A; 2–8 min, 20–90% B and 80–10% A; 8–9 min, 90% B and 10% A; 9–10 min, 90% A and 10% B; column temperature, 40 °C; detection wavelengths, 205 nm, 287 nm, and 330 nm; flow rate, 0.5 mL/min.

### 4.5. Hematoxylin–Eosin (HE) Staining

After fixing the colon tissues using a 4% paraformaldehyde solution, they were embedded in paraffin and sliced into thin sections measuring 5 μm thickness following the process of dehydration. The manufacturer’s instructions were followed for performing the HE staining.

### 4.6. Transcriptomics Analysis of SH Intervention in STC Mouse Colon

The TRIzol method (Invitrogen, CA, USA) was used to extract RNA, followed by treatment with RNase-free DNase I (Takara, Kusatsu, Japan). RNA degradation and contamination were examined on 1% agarose gels to ensure the quality of the extracted RNA. The NanoDrop spectrophotometer (Thermo Scientific, Willmington, DE, USA) was used for quantification and purity analyses. Furthermore, RNA integrity was assessed using Agilent 2100 Bioanalyzer (Agilent Technologies, St. Clara, CA, USA).

We prepared a transcriptome sequencing library using 1.5 μg of RNA per sample as the input material. NEBNext^®^ Ultra™ RNA library prep kit for Illumina^®^ (NEB, Ipswich, MA, USA) was used according to the guidelines of the manufacturer to generate sequencing libraries. Additionally, index codes were incorporated to assign the sequences to their respective samples.

Raw data in the fastq format were obtained after sequencing. Trimmomatic v0.33 was used to generate clean reads, which were then aligned to the mouse reference genome (GCA_001632555.1) using STAR-v2.5.2b. The comparison rate of high-quality reads was typically >90%, which ensured their suitability for subsequent analysis. R (v3.6.0) software was used to generate clustering heatmaps, conduct PCA, and calculate Pearson’s correlation coefficients among the samples. Finally, differential mRNA was subjected to GO and KEGG enrichment analyses using the R package “clusterProfiler.”

### 4.7. 16S rRNA Sequencing and Data Analysis

Microbial genomic DNA was extracted from 24 samples using the PowerSoil DNA isolation kit (MoBio Laboratories, Inc., Carlsbad, CA, USA) [Omega E.Z.N.A.] and stool DNA kit (Omega Bio-Tek, Inc., Norcross, GA, USA). Furthermore, the bacterial 16S rRNA V3-V4 region was amplified with primers 338F (5′-ACTCCTACGGGGGGCAG-3′) and 806R (5′-GACTACHVGGGTWTCTAAT-3′). Polymerase chain reaction (PCR) conditions were as follows: predenaturation at 95 °C for 5 min, denaturation at 95 °C for 45 s, annealing at 55 °C for 50 s, extension at 72 °C for 45 s (28 cycles), and extension at 72 °C for 10 min. Bacterial 16S rRNA amplicon was extracted using 1% agarose gel and Agencourt AMPure XP (Beckman Coulter, Inc., Chaska, MN, USA), purified using NEB Next Ultra II DNA library prep kit (New England Biolabs, Inc., Ipswich, MA, USA), and quantified using the ABI 9700 PCR instrument (Thermo Fisher Scientific, Inc., USA). The purified amplicons were mixed in equimolar amounts and sequenced using the Illumina Hiseq2500 platform (PE250) according to the standard protocol. NEB Next Ultra II DNA library prep kit (New England Biolabs, Inc., USA), a library building kit, was used to construct the microbial diversity sequencing library using Illumina Miseq PE300 (Illumina, Inc.). Paired-end sequencing was performed using a high-throughput sequencing platform (USA).

Trimmatic was used for the quality control analysis of the fastq data. For Trimmatic, a sliding window strategy was employed, with a window size of 50 bp, an average quality value of 20, and a minimum reserved sequence length of 120. During splicing with Pearl (v0.9.6), sequences <230 bp were removed using Vsearch (v2.7.1). The minimum overlap was set to 10 bp and the mismatch rate was set to 0.1. In addition, the UCHIME method was employed to compare and eliminate chimeric sequences. Operational taxonomic unit (OTU) clustering was performed using high-quality sequences with the help of the Vsearch (v2.7.1) UPARSE algorithm, and valid labels were assigned according to the sequence similarity threshold of ≥97% to the same classification units (OTUs). The representative sequence was the tag sequence with the highest abundance within each OTU cluster. Subsequently, Silva138 used the BLAST algorithm for species classification annotation. QIIME (v2.0.0) was used to analyze the α-diversity index (including Chao1, Simpson, Shannon, Pielou_e, Observated_species, Faith_pd, and Goods. coverage), and the Wilcoxon rank test in the R package “ggpubr” (0.4.0) was used to compare the differences between α-diversity indices of the groups. Values at *p* < 0.05 were considered statistically significant. Species composition bar charts were analyzed using R (v3.6.0) based on species annotation and relative abundance results. Based on the weighted uniface distance, the beta diversity distance matrix was calculated using QIIME (v2.0.0) clustering heatmaps were generated and principal coordinate analysis was performed using R (v3.6.0).

### 4.8. Metabonomics Analysis

To start the procedure, 25 mg of the sample was precisely weighed and placed into an EP tube. Then, 500 μL of an extract solution comprising methanol, acetonitrile, and water in a ratio of 2:2:1 was added to the tube. The samples underwent homogenization at a frequency of 35 Hz for 4 min, followed by sonication for 5 min in an ice water bath. This dual-step process of homogenization and sonication was repeated thrice to ensure comprehensive mixing. Subsequently, the sample was centrifuged at 12,000× *g* rpm (RCF = 13,800× *g*, R = 8.6 cm) for 15 min at a temperature of 4 °C. The resulting supernatant was meticulously transferred into a fresh glass vial and prepared for LC/MS analysis. In order to generate the quality control (QC) sample, an equal volume of the supernatant from each sample was combined.

UHPLC (Vanquish, Thermo Fisher Scientific) and Orbitrap Exploris 120 mass spectrometer (Orbitrap, Thermo Fisher Scientific) were utilized for high-performance liquid chromatography (HPLC) separations. The UHPLC system employed was an Acquity UHPLC system (Acquity LC, Waters) equipped with a Waters UPLC column (Acquity UPLC BEH Amide 1.8 µm, 2.1 × 100 mm, Waters, Milford, MA, USA). Mobile phase A consisted of 25 mM ammonium acetate and 25 mM ammonium hydroxide in water, while mobile phase B was 100% ACN. The flow rate was set at 0.5 mL/min, and the injection volume was 2 µL. The samples were kept at 4 °C in the autosampler.

To obtain MS/MS spectra, the Orbitrap Exploris 120 mass spectrometer was employed in the Information Dependent Acquisition (IDA) mode, controlled by the Xcalibur acquisition software (Version:4.4, Thermo). In this mode, the software continuously assessed the full scan MS spectrum. The ESI source conditions were set as follows: sheath gas flow rate of 50Arb, auxiliary gas flow rate of 15Arb, capillary temperature of 320 °C, full MS resolution of 60,000, MS/MS resolution of 15,000, collision energy SNCE of 20/30/40, and spray voltage of 3.8 kV (positive) or −3.4 kV (negative).

### 4.9. Immunofluorescence Double-Label Staining Analysis

Fluorescence homologous double-labeling staining was performed on the paraffin sections. These sections were first dewaxed in water, followed by antigen repair and circling. The sections were incubated with the primary antibody AQP1 (dilution ratio 1:5000), followed by incubation with the horseradish peroxidase (HRP)-labeled secondary antibody. The corresponding tissue-specific antigen (TSA) dye was added, followed by microwave treatment and the addition of the second type of first antibody MUC2 (dilution ratio 1:500). The corresponding secondary antibody was added, followed by DAPI (4′,6-diamidino-2-phenylindole) re-staining of the nucleus, spontaneous fluorescence quenching, sealing, and image visualization, where AQP1 was pink and MUC2 was red.

The paraffin-embedded sections were stained with a fluorescent dye, followed by dewaxing with water, antigen retrieval, and circumcision. The primary antibody SOD1 (dilution ratio 1:100) was added, followed by the addition of the corresponding HRP-labeled secondary antibody and TSA dye. The sections were then subjected to microwave treatment. The corresponding secondary antibody was added, the nuclei were re-stained with DAPI, autofluorescence quenching was performed, slides were mounted, and images were observed, wherein SOD1 was red.

### 4.10. Western Blotting

Tissue samples were used to extract total protein using the RIPA lysis buffer (G2002, Servicebio, Wuhan, China). The protein concentrations were determined using the BCA protein assay kit (G2026, Servicebio, Wuhan). Electrophoresis was conducted on a 10% sodium dodecyl sulfate-polyacrylamide gel, and the resulting proteins were subsequently transferred onto polyvinylidene fluoride (PVDF) membranes. The membranes were then incubated with primary antibodies against AQP1 (1:1000), SOD1 (1:1000), and MUC2 (1:1000) overnight at 4 °C. HRP-labeled secondary antibodies were incubated at room temperature for 2 h. The PVDF membranes were washed 5 times with Tris-buffered saline tween (TBST) and developed using an electrochemiluminescence reagent.

### 4.11. Superoxide Dismutase (SOD) Levels

The SOD levels in mouse serum were measured using the SOD assay kit (A001-3-1, Nanjing Jiancheng Biotechnology Research Institute, Nanjing, China) following the manufacturer’s instructions.

### 4.12. Quantitative Real-Time PCR

The quantitative analysis of a specific DNA sequence in the test sample is commonly performed using fluorescence quantitative PCR with the aid of internal or external standards. Two main methods are typically employed: the non-specific approach utilizing the DNA-binding dye SYBR Green I, and the specific method involving Taqman hydrolysis probes. In this particular experiment, the non-specific SYBR Green I dye method was utilized. SYBR Green I is a dye that binds to the minor grooves of all dsDNA double helices and is excited by green light. Initially emitting weak fluorescence in its free form, the fluorescence of SYBR Green I significantly intensifies upon binding to double-stranded DNA. The fluorescence signal strength of SYBR Green I is directly correlated with the amount of double-stranded DNA present, facilitating the quantification of double-stranded DNA in the PCR system based on fluorescence signals. Reverse transcription involves performing reverse transcription amplification using the QuantiNova reverse transcription kit for qPCR. The cDNA products obtained are then diluted two-fold and used as templates for qPCR amplification with Qiagen ArtiCanCEO SYBR qPCR Mix, followed by on-machine detection.

### 4.13. Molecular Docking

In this study, molecular docking was conducted to investigate the potential binding interactions between the components of SH and specific target proteins. The two-dimensional structures of the initial seven core compounds were obtained from PubChem and prepared by adding charges and flexible bonds. These structures were then visualized using AutoDock Tools (v1.5.6) to ensure accurate representation. Crystal structures of the target proteins were sourced from Protein Data Bank v14 and processed to remove water molecules and impurities using PyMOL. Hydrogen atoms and charges were added to the protein structures using AutoDock Tools for a more realistic simulation. Subsequently, three-dimensional grid boxes necessary for the molecular docking simulations were meticulously generated with AutoDock Tools and visualized using AutoDock Vina (v1.1.2). The docking results were thoroughly analyzed and interpreted with the assistance of PyMOL and Discovery Studio 2020 to gain insights into the potential binding mechanisms between SH components and the target proteins.

### 4.14. Statistical Analysis

The statistical analysis was conducted using SPSS Statistics 19. The results were expressed as the mean ±standard deviation. Differences among two or more groups were analyzed using a one-way analysis of variance and Tukey’s post-test. On the other hand, differences between the two groups were analyzed using a t-test. Statistical significance was defined as *p* < 0.05.

## 5. Conclusions

In conclusion, the analysis of SH composition identified a total of 504 compounds. SH was quantitatively analyzed by HPLC by comparing it with the corresponding standards, and chlorogenic acid (1.028 mg/g), astragalin (0.120 mg/g), rutin (0.240 mg/g), sesamin (0.837 mg/g), sesamolin (0.266 mg/g), α-linolenic acid (0.397 mg/g), and linoleic acid (2.164 mg/g). This study substantiates the efficacy of SH in alleviating STC. SH demonstrates the potential to enhance various aspects of intestinal function, including intestinal propulsion, stool characteristics, and mucosal barrier integrity. The underlying mechanisms involve the regulation of aquaporins, restoration of the intestinal mucosal barrier, and modulation of antioxidant levels. Additionally, SH exhibits the capability to rebalance intestinal flora, further contributing to its therapeutic effect against STC. Future research endeavors should validate the identified therapeutic targets and delve deeper into the molecular and cellular mechanisms involved. This study sheds light on a novel and comprehensive approach through which SH can effectively address the complexities of STC.

## Figures and Tables

**Table 1:** The main components of SH identified by HPLC.

Compound Name	Retention Time (min)	Linear Regression Equation	R^2^	Content (mg/g)
Chlorogenic acid	1.756	y = 96.398x + 0.1338	0.999	1.028
Astragalin	3.931	y = 69.121x − 0.1113	0.999	0.120
Rutin	3.573	y = 42.638x − 0.0621	0.999	0.240
Sesamin	6.842	y = 46.262x − 0.0258	1.000	0.837
Sesamolin	7.117	y = 43.919x + 0.0459	0.999	0.266
α-linolenic acid	9.027	y = 52.997x + 0.0994	0.999	0.397
Linoleic acid	9.569	y = 53.942x + 0.361	0.999	2.164

**Table 2:** The binding energy values of the core compounds of SH and the core targets.

Gene	PDB ID	Compound	PubChem CID	Docking Score (kcal/mol)
AQP1	1h6i	Chlorogenic acid	1,794,427	−6.952
AQP1	1h6i	Rutin	5,280,805	−8.183
MUC2	7qcn	Linolenic acid	5,282,457	−7.277
SOD1	3re0	Linoleic acid	5,280,450	−7.047

## Data Availability

Dates are contained within this article and Supplementary Materials.

## Supplementary Materials

The following supporting information can be downloaded at: https://www.mdpi.com/article/10.3390/molecules29081713/s1, Table S1: SH component analysis and identification results.
